# Towards a harmonized European surveillance for dietary and physical activity indicators in young and adult populations

**DOI:** 10.1093/eurpub/ckac061

**Published:** 2022-11-29

**Authors:** Antje Hebestreit, Stefanie Do, Maike Wolters, Gert B M Mensink, Lina Garnica-Rosas, Karim Abu-Omar, Sven Messing, Agnieszka Neumann-Podczaska, Katarzyna Wieczorowska-Tobis, Nanna Lien, Isobel Stanley, Wolfgang Ahrens, Celine Murrin

**Affiliations:** Department of Epidemiological Methods and Etiological Research, Leibniz Institute for Prevention Research and Epidemiology—BIPS, Bremen, Germany; Department of Epidemiological Methods and Etiological Research, Leibniz Institute for Prevention Research and Epidemiology—BIPS, Bremen, Germany; Faculty of Mathematics and Computer Science, University of Bremen, Bremen, Germany; Department of Epidemiological Methods and Etiological Research, Leibniz Institute for Prevention Research and Epidemiology—BIPS, Bremen, Germany; Department of Epidemiology and Health Reporting, Robert Koch Institute, Berlin, Germany; Department of Epidemiology and Health Reporting, Robert Koch Institute, Berlin, Germany; Department of Sport Science and Sport, Friedrich-Alexander University Erlangen-Nürnberg, Erlangen, Germany; Department of Sport Science and Sport, Friedrich-Alexander University Erlangen-Nürnberg, Erlangen, Germany; Department of Geriatrics and Gerontology, Poznan University of Medical Sciences, Poznan, Poland; Department of Geriatrics and Gerontology, Poznan University of Medical Sciences, Poznan, Poland; Department of Nutrition, Institute of Basic Medical Sciences, University of Oslo, Oslo, Norway; School of Public Health, Physiotherapy and Sports Science, University College Dublin, Dublin, Ireland; Department of Epidemiological Methods and Etiological Research, Leibniz Institute for Prevention Research and Epidemiology—BIPS, Bremen, Germany; School of Public Health, Physiotherapy and Sports Science, University College Dublin, Dublin, Ireland

## Abstract

**Background:**

The Policy Evaluation Network proposes a consolidated approach to measure comparable health indicators across European health surveillance systems to evaluate effectiveness of policy action.

**Methods:**

In a stepwise approach, questionnaire items used by the systems for measuring diet and physical activity data to describe health indicators were identified based on their validity, reliability, and suitability to monitor achievement of health recommendations. They were collated to unified questionnaire modules and discussed bilaterally with representatives of these systems to explore barriers and facilitators for implementation. Also, establishment of a methodological competence platform was proposed, in which the surveillance and monitoring systems agree on the priorities and common quality standards for the harmonization process and to coordinate the integration of questionnaire modules into existing systems.

**Results:**

In total, seven questionnaire modules were developed, of which two diet and two physical activity modules were proposed for implementation. Each module allows measurement of data reflecting only partial aspects of national and WHO recommendations related to diet and physical activity. Main barriers were the requirements of systems to monitor temporal trends and to minimize costs. Main facilitator for implementation was the systems’ use of questionnaire items that were comparable to the unified modules. Representatives agreed to participate in a methodological competence platform.

**Conclusion:**

We successfully took first steps in the realization of the roadmap towards a harmonization of European surveillance by introducing unified questionnaire modules allowing the collection of comparable health indicators and by initiating the establishment of a competence platform to guide this process.

## Introduction

Healthy lifestyles are integral to achieving the Sustainable Development Goals (SDGs).^1^ To evaluate the effectiveness of policies promoting healthy lifestyles, the European member states depend on the regular supply of data on the population’s health and related behaviours. The collection of data describing health behaviour indicators should ideally be carried out in a harmonized manner.^2^^,^^3^ The ultimate aim is to improve data harmonization in a concerted approach, ensuring the comparability of indicators between the regional, national, and international surveillance systems while maintaining the integrity of the participating surveillance systems.^4^ If possible, the harmonization process is guided by an overarching methodological competence platform, similar to the structure proposed by EuroDish^5^; it involves representatives of surveillance and monitoring systems and research institutions and intends to connect state-of-the-art research with cross-country health monitoring. The basis for such an approach was developed by the Determinants of Diet and Physical Activity (DEDIPAC) Consortium,^6^ including a participatory process with representatives of international and regional European surveillance systems. Systems were identified through an inventory^3^ and deemed suitable if they either provided state-of-the-art instruments to measure physical activity and dietary behaviours or had already created a pan-European infrastructure. The Joint Programming Initiative ‘A Healthy Diet for a Healthy Life’ funded Policy Evaluation Network (PEN)^7^ proceeds with this roadmap to establish the stepwise alignment of European surveillance systems, covering different age groups: The WHO European Childhood Obesity Surveillance Initiative (COSI; children 6–9 years),^8^ WHO Health Behaviour in School-Aged Children (HBSC; adolescents 11, 13, and 15 years),^9^ WHO STEPwise approach to Surveillance (STEPS; adult population),^10^ Nordic Monitoring of Diet, Physical Activity and Overweight (NORMO; young and adult population aged 7–12 years and 18–65 years),^11^ and the European Health Interview Survey (EHIS; adolescent and adult population from 15 years onwards).^12^ This publication describes the first steps of the harmonization roadmap^6^:

- The development of short and uniform questionnaire modules for use in future waves of surveillance systems;
- The barriers and facilitators to their implementation;
- The establishment of a methodological competence platform to coordinate and sustain the methodological advancement and harmonization across the systems.

## Methods

### Development of harmonized questionnaire modules

Harmonized questionnaire modules were developed in a four-step process; step one, two and three were previously described.^13^^,^^14^ In the first step, PEN researchers identified key indicators for dietary behaviour, physical activity and sedentary behaviours from: (i) published systematic literature research (e.g. from the DEDIPAC project),^15–17^ (ii) public health frameworks with a focus on health promotion and obesity prevention, such as the Global Action Plan on Physical Activity or the International Network for Food and Obesity/non-communicable diseases Research, Monitoring and Action Support,^17–20^ and (iii) the European Core Health Indicators.^21^ During a Delphi-like expert consultation, key indicators were then prioritized in three stages, considering their relevance for assessing achievement of policy goals, like the SDGs,^1^ and their current or potential adoption by European surveillance systems to evaluate effectiveness of policy action.^13^ The consulted panel included 25 PEN researchers and 15 external experts on monitoring health behaviour indicators at the European level: WHO, OECD, European Commission, World Cancer Research Fund and representatives of the European surveillance and monitoring systems identified in the DEDIPAC inventory.^3^

In the second step, prioritized indicators were assigned to different levels of the socio-ecological model: Policy, community, organizational, interpersonal, and individual level.^22^

In the third step, these indicators were mapped onto variables provided by 17 monitoring and surveillance systems.^14^ Included were systems providing data suitable to describe the indicators, and information on previous survey dates, geographical coverage, and data availability. Based on these criteria, the surveillance systems from the DEDIPAC inventory^3^ were identified and complemented by, e.g. Eurobarometer, WHO Global Nutrition Policy Review, WHO NCD Country Capacity Survey, and the EU Physical Activity Monitoring Framework. Survey databases, handbooks, and questionnaires used in the most recent survey waves were systematically searched for variables that can describe the indicators on the priority list. Not all prioritized indicators could be matched with variables, explaining existing gaps in upstream level data.^14^

In the fourth step, the focus of this paper, we grouped policy level indicators in domains^13^ and selected the top six domains for which indicators of high priority were available at the socio-ecological levels. These six domains were developed further and focused on diet and physical activity rather than sedentary behaviour indicators, which were previously ranked lowest. Questionnaire items used to measure the respective indicators were identified, considering their validity, reliability, and their suitability to measure adherence to the WHO recommendations as selection criteria (Supplementary table S1). Robustness of items in a cross-country context was ensured by the selection of items from the systems’ established instrument catalogues. Aligning the identified questionnaire items to the domain-specific indicators originated sets of unified questionnaire modules: **S**elected **I**nstruments for **M**ultilevel **P**o**L**icy and impact **E**valuation (**SIMPLE**) modules.^23^ In total, seven SIMPLE modules were developed: Food Provision, Food Promotion, and Food Prices as well as Physical Activity Recommendations, Cycling and Walking, and Physical Activity at (Primary or Secondary) School. To improve monitoring of socio-economic disparities,^9^ an additional equity module was developed for collecting inequality indicators for dietary behaviour.

### Consultation process

Key representatives of five multi-national surveillance systems (COSI, HBSC, NORMO, EHIS, and STEPS) were consulted in bilateral video conferences in 2021 to discuss the feasibility to include one or more of the SIMPLE modules in future data collection waves and to identify possible implementation barriers and facilitators. They were also asked if they were interested in becoming an active member of a methodological competence platform. To keep the first harmonization step focused, only the SIMPLE modules Food Prices and Food Promotion as well as Physical Activity Recommendations and Cycling and Walking were suggested for implementation at this point. The choice fell on those modules with most complete indicators at the socio-ecological levels, leaving the least data gaps. To prepare for the consultation, each representative received the underlying DEDIPAC framework,^6^ the SIMPLE modules, and pre-defined questions to prepare for discussion.

Each consultation concluded with the presentation of the aims and structure of the envisaged methodological competence platform and an invitation to join or to nominate an expert. Consultations were recorded, transcribed and the summaries of main agreements were sent back to the representatives to invite feedback and to obtain final approval.

## Results

### Description of SIMPLE modules

The individual level questionnaire items of the Food Prices and Food Provision SIMPLE modules measure fruit and vegetable intake according to the STEPS questionnaire.^24^ They allow measuring consumption frequency and serving size of fruits and of vegetables separately, which is relevant for monitoring the WHO recommendations (400 g of fruit and vegetable/day or five portions/day).^25^ Some indicators were used in several modules; for instance, fruit and vegetable consumption was highly ranked and therefore prioritized as an indicator of healthy dietary behaviour in both the Food Prices and Food Provision modules.

**Figure 1:**
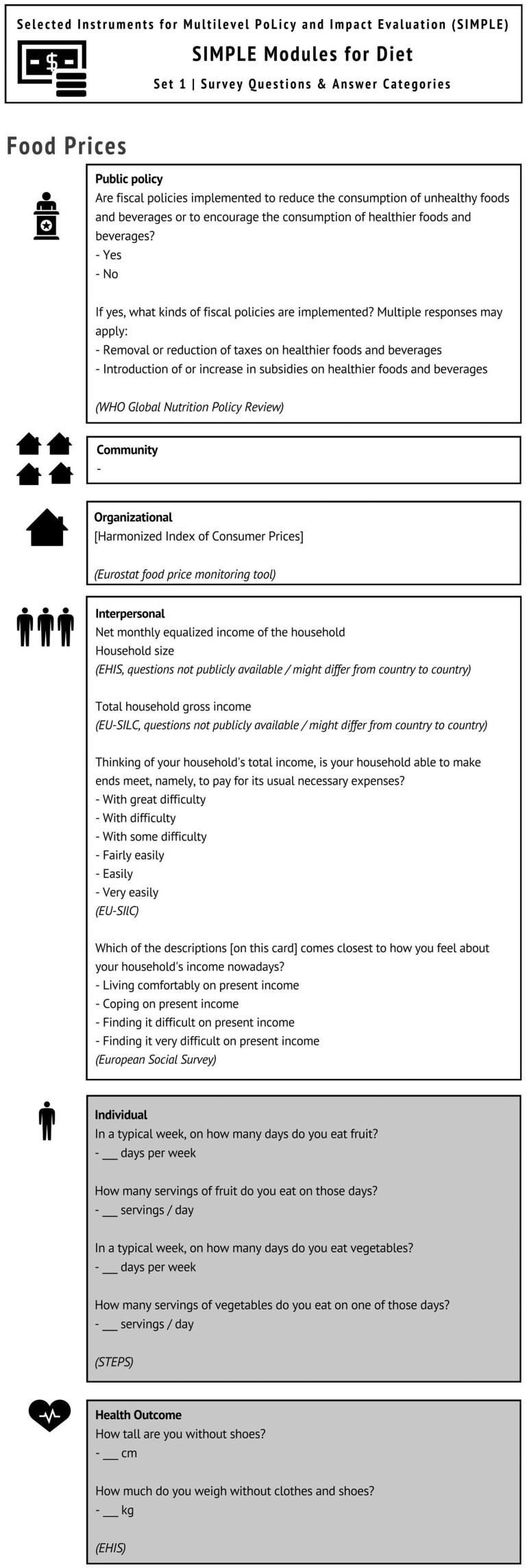
Content of SIMPLE module: Food Prices (Example)

The individual level questionnaire items of the Food Promotion module measure consumption frequency of sugar-sweetened beverages (SSBs) and ultra-processed snack foods from HBSC and COSI, respectively^8^^,^^9^ (table 1). The Food Prices module is presented as an example (figure 1).

**Table 1:** Detailed information on SIMPLE modules for dietary behaviour

Level	Indicator	Survey question [and answer categories]	EU monitoring system/database	A Data gathered from…B ValidationC Proportion of individuals meeting WHO recommendation(s)
**Food prices**				
Public policy	Taxes or levies on unhealthy foods and beverages (e.g. sugar-sweetened beverages, foods high in nutrients of concern) are in place and increase the retail prices of these foods to discourage unhealthy food choices where possible.	Are fiscal policies implemented to reduce the consumption of unhealthy foods and beverages or to encourage the consumption of healthier foods and beverages? [Yes/No] If yes, what kinds of fiscal policies are implemented? Multiple responses may apply: [- Removal or reduction of taxes on healthier foods and beverages] [- Introduction of or increase in subsidies on healthier foods and beverages]	WHO Global Nutrition Policy Review	A National level experts B –
Community	–	–	–	–
Organizational	Relative/absolute cost of healthy and unhealthy foods^a^	[Harmonized Index of Consumer Prices]	Eurostat food price monitoring tool	A Eurostat B –
Interpersonal	Relative household income (household income/household size)	- Net monthly equalized income of the household (Total net monthly income of the household/equivalent household size) - Household size (number of persons living in household, including the respondent)	EHIS (questions not publicly available/might differ from country to country)	A Population (adults) B –
		[Depends on national questionnaire] Total household gross income	EU-SILC (questions not publicly available/might differ from country to country)	A Population (adults) B –
	Financial strain	Thinking of your household’s total income, is your household able to make ends meet, namely, to pay for its usual necessary expenses? [With great difficulty, With difficulty, With some difficulty, Fairly easily, Easily, Very easily]	EU-SILC	A Population (adults) B –
		Which of the descriptions (on this card) comes closest to how you feel about your household’s income nowadays? [- Living comfortably on present income - Coping on present income - Finding it difficult on present income - Finding it very difficult on present income]	European Social Survey	A Population B –
Individuals	Fruit intake, number of portions per day	In a typical week, on how many days do you eat fruit^b^? [___ days per week] How many servings of fruit^b^ do you eat on one of those days? [___ servings/day]	STEPS	A Population (adults) B^26^^d^ C^26^^d^
	Vegetable intake, number of portions per day	In a typical week, on how many days do you eat vegetables^c^? [___ days per week] How many servings of vegetables^c^ do you eat on one of those days? [___ servings/day]	STEPS	A Population (adults) B^26^^d^ C^26^^d^
Health outcome	BMI	How tall are you without shoes? -___ cm How much do you weigh without clothes and shoes? -___ kg	EHIS	A Population (adults) B^27^^,^^28^ C –
**Food promotion (children and adolescents)**				
Public policy	Governmental policies are implemented to restrict commercial marketing (including sponsorship, promotion and advertisement) of unhealthy foods and beverages to children, including adolescents, in settings where children gather (e.g. pre-schools, schools, sports clubs and facilities and cultural events).^a^	Are school health and nutrition policies, programs or related standards being implemented? [Yes/No]	WHO Global Nutrition Policy Review	A National level experts B –
Community	Exposure to food adverts for unhealthy food and beverages through all media and marketing channels.^a^	Are measures to regulate or guide marketing of food and non-alcoholic beverages to children being implemented? [Yes/No] For which communication channels, settings and contexts are the measures mandatory or voluntary? [- TV, - Radio - Advertising (in streets and stores) - Social Media - Apps - Sponsorship - Promotions - Give-aways - Using celebrities] Has any work been done to determine the extent and nature of food marketing in your country (for example through a study or survey)? [Yes/No]	WHO Global Nutrition Policy Review	A National level experts B –
Organizational	School Food Environment	- Which of the following kinds of foods or beverages can pupils obtain on the school premises, excluding lunch provided by the school? Please tick all items that apply. [- Water: (free | paid | N/A) - Tea: (free | paid | N/A) - 100% fruit juices: (free | paid | N/A) - Fruit juices or other non-carbonated drinks: (free | paid | N/A) - Flavoured milk: (free | paid | N/A) - Hot drinks (cocoa, tea, coffee with milk) : (free | paid | N/A) - Fruit: (free | paid | N/A) - Vegetables: (free | paid | N/A) - Sweet snacks (e.g. chocolate, sugar confectionery, cakes, breakfast and/or cereal bars, sweet biscuits and/or pastries): (free | paid | N/A) - Ice-cream: (free | paid | N/A) - Savoury snacks (e.g. potato crisps, salted popcorn, salted nuts, savoury biscuits and/or pretzels): (free | paid | N/A) - Carbonated (soft) drinks: (free | paid | N/A)] - Does your school have a canteen? - [Yes/No] - Does your school have a shop or cafeteria where foods or beverages can be purchased? [Yes/No] - Does your school have vending machines where children are allowed to purchase foods or beverages (other than water, fruits and vegetables)? [Yes/No]	COSI	A Population (school administration) B –
Interpersonal	–	–	–	–
Individuals	Sugar-sweetened beverages, glasses per day	How many times a week do you usually drink …? Coke or other soft drinks that contain sugar^e^ [___ times per week] [___ glasses per occasion]	HBSC	A Population (adolescents) B^29^^,^^30^ C –
	Consumption frequency/day of ultra-processed snack food*	Over a typical or usual week, how often do you eat the following kinds of foods: savoury snacks^f^, sweet snacks^g^? [Never, <1per week, some days (1–3), Most days (4–6), Every day]	COSI	A Population (parents of primary school children) B – C –
Health outcome	BMI	How tall are you without shoes? -___ cm How much do you weigh without clothes and shoes? -___ kg	EHIS	A Population (adults) B^27^^,^^28^ C –
**Food promotion (adults)**				
Public policy	Governmental policies are implemented to support social marketing and fund campaigns to promote healthy and sustainable eating.	Are media campaigns on healthy diet and nutrition conducted? [Yes/No]	WHO Global Nutrition Policy Review	A National level experts B –
Community	–	–	–	–
Organizational	–	–	–	–
Interpersonal	–	–	–	–
Individuals	Sugar-sweetened beverages, glasses per day	How many times a week do you usually drink …? Coke or other soft drinks that contain sugar^e^ [___ times per week] [___ glasses per occasion]	HBSC	A Population (adolescents) B^29^^,^^30^ C –
	Consumption frequency/day of ultra-processed snack food^a^	Over a typical or usual week, how often do you eat the following kinds of foods: savoury snacks^f^, sweet snacks^g^? [Never, <1per week, some days (1–3), Most days (4–6), Every day]	COSI	A Population (parents of primary school children) B – C –
Health outcome	BMI	How tall are you without shoes? -___ cm How much do you weigh without clothes and shoes? -___ kg	EHIS	A Population (adults) B^27^^,^^28^ C –

a Only partial fit of indicator and survey question.

b Serving size: 1 apple, 1 banana, 1 orange, ½ cup cooked or chopped fruit (80 g), ½ cup fruit juice. Examples: fruit and berries include fresh, frozen, canned, glassed/potted etc.; e.g. an apple, an orange, a banana, a bunch of grapes, a plate of strawberries or fruit and berries that are part of porridge, fruit stew, or fruit salad etc.^24^

c Serving size: 1 cup of raw green leafy vegetables (spinach, salad), ½ cup other vegetables, cooked or chopped raw (tomatoes, carrots, pumpkins, corn, Chinese cabbage, fresh beans, onion, etc.), ½ cup vegetable juice. Examples: vegetables, pulses and/or root fruits include fresh, frozen, canned, glass/potted etc.; e.g. carrots, tomatoes, cucumber, broccoli, peppers, salad, beans, chick peas, lentils, beetroot, celery and parsnip.^24^

d Validated questions different compared with those of STEPS, including time frame and using answer categories.

e Examples: energy drinks, red bull, ice tea,…^11^

f Examples: potato crisps, salted popcorn, salted nuts, savoury biscuits and/or pretzels).^11^

g Examples: chocolate, sugar confectionery, cakes, breakfast and/or cereal bars, sweet biscuits, pastries chocolate, candy, biscuits, tart,…^11^^,^^31^

The individual level questionnaire items for the Physical Activity Recommendations and Cycling and Walking SIMPLE modules were taken from EHIS^12^ and measured data that allow for reflecting only partial aspects of the current WHO recommendations, published in 2020^32^ (table 2).

**Table 2:** Detailed information on SIMPLE modules for physical activity

Level	Indicator	Survey question [and answer categories]	EU monitoring system/database	A Data gathered from…B ValidationC Proportion of individuals meeting WHO recommendation(s)
**Physical activity recommendations**				
Public policy	National recommendations on physical activity for health	Are there national guidelines which provide recommended levels of physical activity for the population or a specific segment of the population? [Yes, No, Don’t know] If yes, are there guidelines specifically addressing any of the following age groups: - Children under 5: [Yes | No | Don’t Know] - Children and adolescents (ages 5–19 years): [Yes | No | Don’t Know] - Adults: [Yes | No | Don’t Know] - Older adults: [Yes | No | Don’t Know]	WHO NCD Country Capacity Survey	A National level experts B –
Community	Proportion of people aware of physical activity programmes organized by the community^a^	To what extent do you agree or disagree with the following statement about sport and physical activity? The area where you live offers you many opportunities to be physically active. [Agree/Disagree]	Eurobarometer	A Population (adults) B –
Organizational	Settings included in the delivery of specific health-enhancing physical activity actions	Considering all (…) key physical activity policy documents (…), please indicate which settings are included for the delivery of specific Health-Enhacing Physical Activity actions. [- Pre-schools/kindergarten - Primary schools - Secondary/high schools - Colleges/universities - Primary health care - Clinical health care (e.g. hospitals) - Workplace - Older adult/senior services - Sport and recreation - Transport - Tourism - Environment - Urban design and planning - Community]	HEPA PAT	A National level experts B –
Interpersonal	Proportion of people who see others being active in their neighbourhood^a^	Earlier you said you engage in sport or another physical activity, vigorous or not. Where do you do this? [- In a park, outdoors, etc. - At home - On the way between home and school, work or shops - At a health or fitness centre - At a sport club - At school or university - Elsewhere (spontaneous) - Don’t know]	Eurobarometer	A Population (adults) B –
Individuals	Total time spent with physical activity per week	In a typical week, on how many days do you carry out sports^b^, fitness^c^ or recreational^d^ (leisure) activities for at least 10 min^e^ continuously? [___ days per week]	EHIS	A Population (adults) B^33^^f^ C –
		How much time in total do you spend on sports^b^, fitness^c^ or recreational^d^ (leisure) physical activities in a typical week? [- ___ hours per week - ___ minutes per week]	EHIS	A Population (adults) B^33^^f^ C –
**Cycling and walking**				
Public policy	Government supports the incorporation of walking and cycling infrastructure in urban, rural and transport plans^a^	Are the European Guidelines for Improving Infrastructures for Leisure-Time Physical Activity applied systematically to develop leisure-time infrastructures in your country? [- Implemented - Foreseen in the next 2 years - Not implemented]	EU/WHO HEPA Monitoring Framework survey	A National level experts B –
Community	Availability and quality of cycling networks/paths/amenities; cycle-friendly infrastructure	[User generated content, e.g. bicycle map, hiking map, wheelchair user map]	Open Street Maps	A Users B Validity differs from region to region due to the user-generated content and the differences in user numbers and their activity^34^
Organizational	–	–	–	–
Interpersonal	–	–	–	–
Individuals	Time spent walking in order to get to and from places in a typical week	In a typical week, on how many days do you walk for at least 10 min continuously in order to get to and from places? [___ days per week]	EHIS	A Population (adults) B^33^^g^ C –
		How much time do you spend walking in order to get to and from places on a typical day? [- 10–29 min per day - 30–59 min per day - 1 h to less than 2 h per day - 2 h to less than 3 h per day - 3 h or more per day]	EHIS	A Population (adults) B^33^^g^ C –
	Time spent cycling in order to get to and from places in a typical week	In a typical week, on how many days do you bicycle for at least 10 min continuously to get to and from places? [___ days per week]	EHIS	A Population (adults) B^33^^h^ C –
		How much time do you spend bicycling to get to and from places on a typical day? [- 10–29 min per day - 30–59 min per day - 1 h to less than 2 h per day - 2 h to less than 3 h per day - 3 h or more per day]	EHIS	A Population (adults) B^33^^h^ C –

a Only partial fit of indicator and survey question.

b Examples (sports): ball games, athletics, competitive bicycling, running, swimming, etc.^35^

c Examples (fitness): endurance training, strength exercise, flexibility training, etc.^35^

d Examples (recreational activity): nordic walking, brisk walking, ball games, jogging, bicycling, swimming, aerobics, rowing, badminton, etc.^35^

e The time frame of ‘at least 10 minutes’ is no longer recommended in WHO’s Global Physical Activity Guidelines, i.e. a modification of this survey question might be necessary in future.

f Applicable to moderate-to-vigorous aerobic recreational activity (minutes per day).

g Applicable to walking time (minutes per day).

h Applicable to cycling time (minutes per day).

For some modules like the Food Promotion module, different versions were provided addressing different settings for policy implementation, such as work place (adults) and school (children, adolescents). The individual level questionnaire item for the Physical Activity at School module was separated into two versions, one for primary school children (reported by parents) and one for secondary school children (self-reported). Generally, individual level questionnaire items are self-reported during Computer Assisted Telephone Interviews (e.g. EHIS, NORMO), self-reported using online questionnaires (eSTEPS app), self-completed in the classroom (e.g. HBSC), or proxy-reported by parents/caregivers (e.g. COSI, NORMO). Variables to describe organizational, community, and policy level indicators are available from national routine monitoring data sets. The SIMPLE modules were developed to facilitate pragmatic implementation of short questionnaire items and not all were validated against a standard measure, e.g. accelerometers. However, a study on the psychometric properties of the EHIS-PAQ, which is comparable to the questionnaire items we selected for the SIMPLE modules, revealed strong to moderate reliability and poor to moderate validity depending on the sub-domains measured^33^ (Supplementary table S1). The diet questionnaire items were compared against, e.g. a 7-day food diary and a 74-item FFQ. They revealed a strong reliability for fruit, vegetable and SSB intake as well as moderate (for fruits), fair (for vegetables) and strong (for SSB) validity. The domains and sub-domains of the dietary behaviour and physical activity SIMPLE modules differ in nature, as indicators for both behaviours are operationalized differently across European countries and surveillance systems.^14^ SIMPLE modules are provided on the PEN website.^23^Supplementary tables S2 and S3 provide the remaining modules. A summary of the consultative process including the systems representatives’ opinion is provided in Supplementary table S4.

### Implementation of SIMPLE modules in existing systems and establishment of a methodological competence platform

#### Fundamental considerations

The SIMPLE modules were developed to provide a first core data set of comparable diet and physical activity indicators as well as body height and weight (for calculating the Body Mass Index, BMI). Ideally, individual level questionnaire items of the SIMPLE modules will be used in next survey waves of the surveillance systems. To start the implementation, it is possible to either introduce the individual level questionnaire items only in voluntary modules, in sub-samples, or to pilot the instruments in national surveys during or between the next survey waves without discarding existing instruments.^6^

Each SIMPLE module further facilitates identification of routine monitoring data sets providing data on organizational, community, and policy level indicators relevant for one of the six policy domains and the diet module on equity. Hence, researchers, key stakeholders, and policy-makers can use these monitoring data sources to combine national or international data for upstream indicators with newly measured individual level data. Validity and reliability of the selected questionnaire items were discussed critically with all systems as well as their suitability to monitor achievement of WHO recommendations. The concern that measured data can only reflect certain aspects of the WHO recommendations was raised for both, physical activity and diet modules.

#### Implementation process and methodological competence platform establishment

Implementation of the individual level question of the SIMPLE modules should be supplemented by methodological studies, to further improve and modernize established surveillance questionnaires, because several of them are not ‘fit for purpose’. Questionnaire items that have been introduced decades ago^36^ may require adaptation to the current recommendations, e.g. for physical activity,^32^ or to capture changes in circumstances. As an example, variables like ‘Capacity to afford a meal with meat, chicken, fish (or vegetarian equivalent) every second day’ to measure food insecurity (EU-SILC) may no longer be suitable in times of planetary health diets.^37^

Further studies investigating validity and reliability of questionnaire items in different age groups and—equally important—their suitability to monitor WHO recommendations are needed. As several prioritized indicators could not be matched to variables from existing data sources, the data gaps may be closed with questionnaire items from research studies. To address these methodological challenges, to guide and sustain the harmonization process, and to jointly support and coordinate the necessary methodological developments a methodological competence platform will be established. Besides research institutions the involvement of national and international surveillance systems is desirable. Membership in the methodological competence platform was approved by most representatives (COSI, HBSC, NORMO, STEPS) or will be decided later (EHIS). Platform members meet at regular intervals to agree on the priorities for the harmonization process and common quality standards, to coordinate the necessary action, to propose harmonized surveillance modules for integration into existing systems and to push methodological advancements.^6^

#### Piloting the SIMPLE modules

In most systems, formalized processes for changes or extensions of the questionnaires in use are established including steering committees with working groups or sub-committees: The responsible group/committee typically recommends the voluntary piloting of the individual level questionnaire items (such as SIMPLE modules) during the next national survey to the system’s steering group. If the evaluation of the pilot results shows adequate suitability, and improvement to the system’s data sets, their implementation will be suggested for the core (mandatory) questionnaire.

##### Dietary behaviour

Most surveillance initiatives stated an interest in piloting diet questionnaire items of the Food Prices and Food Promotion modules. This was because they already used the suggested questionnaire item (STEPS, COSI, HBSC) or a comparable instrument, such as a food frequency questionnaire (e.g. NORMO, COSI). Food frequency data can be used to calculate the daily fruit and vegetable intake (g/day) if it includes consumed quantities like in STEPS.^10^ As NORMO assesses food consumption frequency using the same instruments in children and adults, adherence to the WHO recommendation can be evaluated in both age groups without adding a similar question.

##### Physical activity

The individual level questionnaire items of the physical activity SIMPLE modules only use parts of validated instruments since the full instruments were considered to be too lengthy and put a high burden on respondents, i.e. the Global Physical Activity Questionnaire. As the SIMPLE modules were less suitable for assessing all aspects of the WHO recommendation, only one system (NORMO) expressed an interest to include the Cycling and Walking SIMPLE module in future waves, but rather as a proxy for measuring sustainable and climate friendly transportation alternatives than for monitoring physical activity of the population.

##### Body mass index

The BMI was considered as the most appropriate health outcome for the SIMPLE modules. It is either self-reported (HBSC, EHIS, NORMO), or anthropometrically measured in two systems (COSI, STEPS). BMI is assessed in a comparable way by many systems and may thus be considered as an already harmonized indicator across systems and age groups.

#### Perceived facilitators and barriers of implementation of SIMPLE modules

##### Facilitators

In general, the systems’ representatives agreed that harmonizing data collection across countries and age groups is desirable and most representatives expressed interest in implementing one or more SIMPLE modules. Of particular interest were the individual level questionnaire items measuring indicators describing sustainability aspects, such as cycling and walking, or fruit and vegetable intake. The systems’ representatives proclaim that implementation could be facilitated if all surveillance systems compromise on one or more unified questionnaire modules. In addition, ongoing harmonization processes within WHO were mentioned as a facilitator: COSI recently started a process to harmonize questionnaire items of the family questionnaire with HBSC.

##### Barriers

The main concern against the implementation of the SIMPLE modules was the lack of comparability with current data and the objective to monitor temporal trends (all systems). Concerns were also raised regarding the questionnaire length; it is unsuitable to repeat very similar questions and only a limited number of new questions and variables can be added between waves (all systems). Hence, newly introduced instruments need to be broken down to the variable level, with a preference for instruments entailing a small number of variables (EHIS).^38^ Overall, nationally driven interests related to population health surveillance, such as the need to measure sustainability indicators in future waves must be balanced against limited national resources (NORMO).

## Discussion

The DEDIPAC Knowledge Hub developed a roadmap towards a harmonized European surveillance system^3^^,^^6^ and PEN successfully took first steps in its realization.^13^^,^^14^ The SIMPLE modules were proposed for implementation in ongoing surveillance systems during a consultative process. The bilateral consultations revealed a general consensus that the harmonization of data collection is desirable and incorporation of the SIMPLE modules was, in principle, met with approval by most surveillance systems.

### Leveraging the facilitators for implementation

Implementation of the modules should be facilitated by the questionnaire items of SIMPLE modules being derived from established surveillance systems. The gained experiences from the pilot studies may guide further adaptations to be tested again in sub-samples until an improved version is provided on a European level. Further, the tendency of systems to keep the list of indicators as stable as possible for trend analyses bears the risk of hampering innovation. However, for different reasons updates of indicators or new measurements may become necessary for the systems. Such reasons include changes to European health surveys, or new relevant public health topics, like climate friendly transportation alternatives^39^ or sustainable diets.^37^ However, most surveillance systems make strategic decisions to modernize their assessment methods or questionnaires.^40^ This is part of the trade-off between the continuation of indicator measures and keeping them up-to-date.

### Addressing the barriers for implementation

A critical functionality of surveillance is to measure indicators at a certain moment or over a period of time to monitor changes in prevalence of health status, in habitats, populations, and environments,^41^ facilitated by repeated standardized surveys.^39^ The systems’ concern that monitoring trends will be hampered when the new unified questionnaire modules are implemented may be alleviated, as inclusion of questionnaire items without discarding the established systems’ questions will secure the system’s internal integrity and retain their ability to assess temporal trends.^6^

As the surveillance systems are tightly regulated and will only allow a certain percentage of items to be changed between survey waves, the inclusion of new questionnaire items requires a thorough appraisal.^39^ As an example, for EHIS surveys, the European Commission allows for a maximum of 10% change between the required variables, and a maximum increase of 5% in the number of variables between EHIS waves 3 and 4, offering a certain degree of flexibility.^38^ However, any changes in the observed indicator or variable of interest may be explained solely or partly by the change in instruments between survey waves rather than by real changes in health behaviour or health status. This concern may be ruled out as PEN proposed to initially measure indicators using established and uniform questionnaires in parallel, allowing to monitor actual changes. To make sure that these changes are the result of (not) implemented policies, data analysis may apply difference-in-difference design to account for secular trends.^42^ However, changing the instruments requires a clear purpose for the planned use of the gathered information,^43^ also to keep the costs at a minimum. In this regard, we have to acknowledge that including the SIMPLE modules will add costs of collection, storage, and analysis of data, unless questionnaires are shortened in other parts. Thus, existing embedded indicators measured insufficiently by poor instruments are the best candidates for replacement. For example, some instruments currently in use to measure physical activity behaviour in everyday life situations (such as mobility and movement patterns, or sedentary and exercise behaviour) have shown low validity.^44^ The much-needed improvement may be seen in wearable sensors that are increasingly popular in research but not yet implemented in surveillance. Alternatively, valid and robust single-item questions are much shorter compared with established instruments; however, additional methodological studies are required to identify the most reliable and valid question^45^ that also should serve the purpose to monitor the WHO recommendations.^32^

The context and political climate in a country may determine what indicators are prioritized and how they are measured. We are just learning this during the COVID-19 pandemic and the projected acute food insecurity due to the war in Ukraine, that new health threats, demographic change, and inequalities in health and healthcare provision in and between EU member states challenge the national health care systems. Thus, to overcome the most urgent public health problems, systemic and integrative approaches are needed, and require responsive and consistent surveillance systems working across national borders.^46^ This will generate comparable European health data to guide prioritization of health promotion measures, and to raise public and political awareness of the extent of public health problems.^43^

Health status and health behaviours are largely affected by socio-economic disparities and need to be monitored better across countries and age groups. The diet equity module required further enhancement before implementation since its questionnaire items could only be identified for interpersonal and individual level, leaving data gaps for downstream indicators. Also, developing a physical activity module on equity would be highly relevant; however, current surveillance systems do not measure indicators that would allow mapping out such a module.

In summary, PEN successfully took first steps in the realization of the roadmap towards harmonized European surveillance. Future steps in the harmonization process should not only cover methodological advancement of the SIMPLE modules, or implementation of objective measurements, but also expand usage of the SIMPLE modules by additional national and international surveillance systems. Increasing the comparability of data across surveys, age groups, and countries is essential to evaluate effectiveness of policy action and to inform decision making and appropriate public health action. Improving impact evaluation of health policy interventions in turn will pave the way for healthier food and physical activity environments across Europe.

## Supplementary data

Supplementary data are available at *EURPUB* online.

## Supplementary Material

ckac061_Supplementary_Data
