# Supplementary material for: Towards a harmonized European surveillance for dietary and physical activity indicators in young and adult populations
Source: Eur J Public Health. 2022 Nov 29;32(Suppl 4):iv21–31. doi: 10.1093/eurpub/ckac061 (PMC9706124; doi:10.1093/eurpub/ckac061)
Supplement: ckac061_Supplementary_Data [file ckac061_supplementary_data.zip › ckac061_Supplementary_Data/Hebestreit_Monitoring_SupplMat1.docx]

**Supplementary Table 1: Reliability and validity of the individual level indicators**

| **Individual level indicator** | **SIMPLE Module** | **Question in reliability and/or validation study** | **WHO recommendation** | **Reliability^a^** | **Validity^a^** | **Monitoring of WHO recommendations^a^** |
| --- | --- | --- | --- | --- | --- | --- |
| Fruits and Vegetables intake, number of portions per day | Food Prices,  Food Provision,  Equity | Over the last 1 month only^b^, on average, how many serves of fruit did you eat per day?” [None, Less than 1 serving per day, 1 serving per day, 2 servings per day, 3 servings per day, and 4 or more serving per day]  Over the last 1 month only*, on average, how many serves of vegetables did you eat per day?” [None, Less than 1 serving per day, 1 serving per day, 2 servings per day, 3 servings per day, and 4 or more serving per day] | 400g edible fruit and vegetables per day | Strong  (for fruits and vegetables) (1) | Moderate (for fruits) and fair (for vegetables) (1) | Fair agreement (for vegetables) and moderate agreement (for fruits) against 74-item FFQ  (1)^c^ |
| Consumption of sugar sweetened beverages | Food Promotion | Asking how many times a week they ate fruits, vegetables, sweets and chocolates (labeled ‘‘sweets’’), and sweetened soft drinks (cola or other soft drinks that contain sugar).  [Never, < 1/wk, about 1/wk, 2–4d/wk, 5–6d/wk, once every day,or every day, more than once] | Sugar intake of 10% of total energy intake | Strong (5) | Strong (5) | ./. |
|  |  |  |  | ./. | Moderate (4) | ./. |
| Ultra-processed snack food consumption | Food Promotion | ./. |  | ./. | ./. | ./. |
| BMI | Food Prices,  Food Promotion,  Food Provision,  Equity | How tall are you without shoes? in [cm]  └─┴─┴─┘ [cm]  How much do you weigh without clothes and shoes? in [kg]  └─┴─┴─┘ [kg] | for adults:  Underweight: Below 18.5  Normal weight: 18.5–24.9  Pre-obesity: 25.0–29.9  Obesity class I: 30.0–34.9  Obesity class II: 35.0–39.9  Obesity class III: Above 40    for children and adolescents (5-19 years):  Overweight: >+1SD (equivalent to BMI 25 kg/m2 at 19 years)  Obesity: >+2SD (equivalent to BMI 30 kg/m2 at 19 years)  Thinness: <-2sd>  Severe thinness: <> | Strong (12) | Strong (2,3) | ./. |
| Total time spent with physical activity per week | Physical Activity Recommendations | In a typical week, on how many days do you carry out sports, fitness or recreational (leisure) activities for at least 10 minutes continuously?  [___ days per week]  How much time in total do you spend on sports, fitness or recreational (leisure) physical activities in a typical week?  [ -___ hours per week  - ___ minutes per week] | For adults: 150 minutes of moderate-intensity aerobic physical activity throughout the week; aerobic activity performed in bouts of at least 10 minutes duration  For adults: 150 minutes of moderate-intensity aerobic physical activity throughout the week; aerobic activity performed in bouts of at least 10 minutes duration | Strong for moderate-to-vigorous aerobic recreational activity (min/d) (9) | Fair to moderate for moderate-to-vigorous aerobic recreational activity(min/d) (9) | ./. |
| Time spent walking in order to get to and from places in a typical week | Cycling and Walking | In a typical week, on how many days do you walk for at least 10 minutes continuously in order to get to and from places?  [ ___ days per week]  How much time do you spend walking in order to get to and from places on a typical day?  [- 10-29 minutes per day  - 30-59 minutes per day  - 1 hour to less than 2 hours per day  - 2 hours to less than 3 hours per day  - 3 hours or more per day] |  | Moderate for walking time (min/d) (9) | Poor to moderate to high for walking time (min/d) (9) | ./. |
| Time spent cycling in order to get to and from places in a typical week | Cycling and Walking | In a typical week, on how many days do you bicycle for at least 10 minutes continuously to get to and from places?  [ ___ days per week]  How much time do you spend bicycling to get to and from places on a typical day?  [- 10-29 minutes per day  - 30-59 minutes per day  - 1 hour to less than 2 hours per day  - 2 hours to less than 3 hours per day  - 3 hours or more per day] |  | Moderate for cycling time (min/d) (9) | Fair to moderate for cycling time (min/d) (9) | ./. |
| Total time spent with physical activity per week [primary school] | Physical Activity at Primary Schools | ./. | For children and adolescents: at least 60 minutes per day of moderate-to-vigorous intensity, mostly aerobic, physical activity, across the week; incorporate vigorous-intensity aerobic activities, as well as those that strengthen muscle and bone, at least 3 days a week | ./. | ./. | ./. |
| Total time spent with physical activity per week [secondary school] | Physical Activity at Secondary Schools | Over the past 7 days, on how many days were you physically active for a total of at least 60 minutes per day? Please add up all the time you spent in physical activity each day. [0-7 days] |  | Strong (13) | Fair (13) | ./. |
|  |  |  |  | ./. | Moderate (14) | Strong agreement against accelerometry data (14) |

^a^ Own scale for validity, reliability, and suitability to monitor WHO recommendations: 0–0.20 = poor, >0.20–0.40 = fair, >0.40–0.60 = moderate, >0.60 = strong

^b^ Time frame and answer categories slightly different from STEPS

^c^ STEPS evaluates WHO recommendation, especially by asking for servings so the amount can be deciphered
