# Supplementary material for: Towards a harmonized European surveillance for dietary and physical activity indicators in young and adult populations
Source: Eur J Public Health. 2022 Nov 29;32(Suppl 4):iv21–31. doi: 10.1093/eurpub/ckac061 (PMC9706124; doi:10.1093/eurpub/ckac061)
Supplement: ckac061_Supplementary_Data [file ckac061_supplementary_data.zip › ckac061_Supplementary_Data/Hebestreit_Monitoring_SupplMat2&3.docx]

**Supplementary Table 2: Detailed information on SIMPLE modules for dietary behaviour**

| **Food Provision (children and adolescents)** | | | | |
| --- | --- | --- | --- | --- |
| **Level** | **Indicator** | **Survey question**  **[and answer categories]** | **EU Monitoring System / Database** | **A Data gathered from B Validation C Proportion of individuals meeting WHO recommendation(s)** |
| Public policy | The government ensures that there are clear, consistent policies (including nutrition standards) which can be feasibly implemented in schools and early childhood education services for food service activities (canteens, food at events, fundraising, promotions, vending machines etc.) to provide and promote healthy food choices. | Are school health and nutrition policies, programs or related standards being implemented?  [Yes/No] | WHO Global Nutrition Policy Review | A National level experts  B - |
| Community | Exposure to food adverts for unhealthy food and beverages through all media and marketing channels.^a^ | Are measures to regulate or guide marketing of food and non-alcoholic beverages to children being implemented?  [Yes/No]  For which communication channels, settings and contexts are the measures mandatory or voluntary?  [- TV,  - Radio  - Advertising (in streets and stores)  - Social Media  - Apps  - Sponsorship  - Promotions  - Give-aways  - Using celebrities]  Has any work been done to determine the extent and nature of food marketing in your country (for example through a study or survey)?  [Yes/No] | WHO Global Nutrition Policy Review | A National level experts  B - |
| Organisational | School Food Environment | Which of the following kinds of foods or beverages can pupils obtain on the school premises, excluding lunch provided by the school? Please tick all items that apply.  [- Water: (free \| paid \| N/A)  - Tea: (free \| paid \| N/A)  - 100% fruit juices: (free \| paid \| N/A)  - Fruit juices or other non-carbonated drinks: (free \| paid \| N/A)  - Flavoured milk: (free \| paid \| N/A)  - Hot drinks (cocoa, tea, coffee with milk) : (free \| paid \| N/A)  - Fruit: (free \| paid \| N/A)  - Vegetables: (free \| paid \| N/A)  - Sweet snacks (e.g. chocolate, sugar confectionery, cakes, breakfast and/or cereal bars, sweet biscuits and/or pastries): (free \| paid \| N/A)  - Ice-cream: (free \| paid \| N/A)  - Savoury snacks (e.g. potato crisps, salted popcorn, salted nuts, savoury biscuits and/or pretzels: (free \| paid \| N/A)  - Carbonated (soft) drinks: (free \| paid \| N/A)]  Does your school have a canteen?  [Yes/No]  Does your school have a shop or cafeteria where foods or beverages can be purchased?  [Yes/No]  Does your school have vending machines where children are allowed to purchase foods or beverages (other than water, fruits and vegetables)?  [Yes/No] | COSI | A Population  (school administration)  B - |
| Interpersonal | - | - | - | - |
| Individuals | Fruit intake, number of portions per day | In a typical week, on how many days do you eat fruit^b^?  [___ days per week]  How many servings of fruit^b^ do you eat on one of those days?  [___ servings/ days] | STEPS | A Population (adults)  B (1)^d^  C (1)^d^ |
|  | Vegetable intake, number of portions per day | In a typical week, on how many days do you eat vegetables ^c^?  [___ days per week]  How many servings of vegetables^c^ do you eat on one of those days?  [___ servings/ days] | STEPS | A Population (adults)  B (1)^d^  C (1)^d^ |
| Health outcome | BMI | How tall are you without shoes?  └─┴─┴─┘ cm  How much do you weigh without clothes and shoes? └─┴─┴─┘ kg | EHIS | A Population (all age groups) B (2,3) C - |

| **Food Provision (adults)** | | | | |
| --- | --- | --- | --- | --- |
| **Level** | **Indicator** | **Survey question**  **[and answer categories]** | **EU Monitoring System / Database** | **A Data gathered from B Validation C Proportion of individuals meeting WHO recommendation(s)** |
| Public policy | The government ensures that there are clear, consistent policies, which can be feasibly implemented, in other public sector settings for food service activities (canteens, food at events, fundraising, promotions, vending machines, public procurement standards etc.) to provide and promote healthy food choices. | Are there national nutrition policies, strategies or action plans or other policies and plans (e.g. health sector plans, social protection plans, food security strategies) which focus on nutrition or healthy diet?  [Yes/No]  Which nutrition related actions or interventions are included in the policy, strategy or plan?  [Creation of healthy food environment in workplaces:   - In hospitals - Other places (please specify)] | WHO Global Nutrition Policy Review | A National level experts  B - |
| Community | Neighbourhood healthy food availability | - | - | - |
| Organisational | Portion size from manufacturers and food outlets in settings | - | - | - |
| Interpersonal | - | - | - | - |
| Individuals | Fruit intake, number of portions per day | In a typical week, on how many days do you eat fruit^b^?  [___ days per week]  How many servings of fruit^b^ do you eat on one of those days?  [___ servings/ days] | STEPS | A Population (all age groups)  B (1)^d^  C (1)^d^ |
|  | Vegetable intake, number of portions per day | In a typical week, on how many days do you eat vegetables^c^?  [___ days per week]  How many servings of vegetables^c^ do you eat on one of those days?  [___ servings/ days] | STEPS | A Population (adults)  B (1)^d^  C (1)^d^ |
| Health outcome | BMI | How tall are you without shoes?  └─┴─┴─┘ cm  How much do you weigh without clothes and shoes? └─┴─┴─┘ kg | EHIS | A Population (adults) B (2,3) C - |

| **Equity** | | | | |
| --- | --- | --- | --- | --- |
| **Level** | **Indicator** | **Survey question**  **[and answer categories]** | **EU Monitoring System / Database** | **A Data gathered from B Validation C Proportion of individuals meeting WHO recommendation(s)** |
| Public policy | There are processes in place to ensure that population nutrition, health outcomes and reducing health inequalities or health impacts in vulnerable populations are considered and prioritized in the development of all government policies relating to food. | Currently not measured in an ongoing EU surveillance system. | - | - |
| Community | - | - | - | - |
| Organisational | Waste reduction policies for food retail and food service outlets are in place | Currently not measured in an ongoing EU surveillance system. | - | - |
| Interpersonal | Relative household income (household income/household size) | Ask all adults:  We put answers into income bands. Would you tell me which band represents your total personal income before all deductions? Is it... | EU-SILC | A Population B - |
| Individuals | Fruit intake, number of portions per day | In a typical week, on how many days do you eat fruit ^b^?  [___ days per week]  How many servings of fruit^b^ do you eat on one of those days?  [___ servings/ days] | STEPS | A Population (adults)  B (1)^d^  C (1)^d^ |
|  | Vegetable intake, number of portions per day | In a typical week, on how many days do you eat vegetables^c^?  [___ days per week]  How many servings of vegetables^c^ do you eat on one of those days?  [___ servings/ days] | STEPS | A Population (adults)  B (1)^d^  C (1)^d^ |
| Health outcome | BMI | How tall are you without shoes?  cm └─┴─┴─┘  How much do you weigh without clothes and shoes? kg └─┴─┴─┘ | EHIS | A Population (all age groups) B (2,3) C - |

^a^ Only partial fit of indicator and survey question

^b^ Serving size: 1 apple, 1 banana, 1 orange, ½ cup cooked or chopped fruit (80 g), ½ cup fruit juice. Examples: fruit and berries include fresh, frozen, canned, glassed/potted etc.; e.g. an apple, an orange, a banana, a bunch of grapes, a plate of strawberries or fruit and berries that are part of porridge, fruit stew, or fruit salad etc. (6)

^c^ Serving size: 1 cup of raw green leafy vegetables (spinach, salad), ½ cup other vegetables, cooked or chopped raw (tomatoes, carrots, pumpkins, corn Chinese cabbage, fresh beans, onion, etc.), ½ cup vegetable juice. Examples: vegetables, pulses and/or root fruits include fresh, frozen, canned, glass/potted etc.; e.g. carrots, tomatoes, cucumber, broccoli, peppers, salad, beans, chick peas, lentils, beetroot, celery and parsnip. (6)

^d^ Validated questions different compared to those of STEPS, including time frame and using answer categories.

**Supplementary Table 3: Detailed information on SIMPLE modules for physical activity**

| **Physical activity at Primary Schools** | | | | |
| --- | --- | --- | --- | --- |
| **Level** | **Indicator** | **Survey question**  **[and answer categories]** | **EU Monitoring System / Database** | **A Data gathered from...**  **B Validation**  **C Proportion of individuals meeting WHO recommendation(s)** |
| Public policy | Government supports schools to include physical education in school curricula | In your country, is physical education a compulsory school curriculum subject in primary school? | 4^th^ Worldwide Survey of Quality Physical Education | A National level experts  B - |
| Community | Condition of active commuting infrastructure to and from school | In your opinion, how safe are the routes to and from school for your child to walk or ride a bicycle, skateboard or non-motorized scooter?  [(Please circle the appropriate number)  - extremely safe 1 2 3 4 5 6 7 8 9 10 extremely unsafe] | COSI | A Population (parents of primary school children)  B - |
| Organisational | Average curricular physical education time per week | Within the policy framework of the compulsory school programme, how much time is allocated to the physical education curriculum each week in primary schools? | 4^th^ Worldwide Survey of Quality Physical Education | A National level experts  B - |
| Interpersonal | Proportion of young people who receive supervision from educators/teachers to be physically active^a^ | Does your school organise any sport/physical activities at least once a week for primary school children outside school hours?  [- Yes, for all grade levels  - Only to some grade levels (please specify the grade): ________  - No]  Do children attend these organised sport/physical activities?  [- Yes, more than half of the children  - Yes, half or less than half of the children  - No or mostly not (less than a quarter of the children)] | COSI | A Population (parents of primary school children)  B - |
| Individuals | Total time spent with physical activity per week^a^ | In his/her free time, about how many hours per day is your child usually playing actively/vigorously (e.g. running, jumping outside or moving and fitness games inside)?    [Weekdays: Never at all, less than 1 hour per day, about 1 hour per day, about 2 hours per day, about 3 or more hours per day]  [Weekend: Never at all, less than 1 hour per day, about 1 hour per day, about 2 hours per day, about 3 or more hours per day] | COSI^b^ | A Population (parents of primary school children)  B -  C - |

| **Physical Activity at Secondary Schools** | | | | |
| --- | --- | --- | --- | --- |
| **Level** | **Indicator** | **Survey question**  **[and answer categories]** | **EU Monitoring System / Database** | **A Data gathered from...**  **B Validation**  **C Proportion of individuals meeting WHO recommendation(s)** |
| Public policy | Government supports schools to include physical education in school curricula | In your country, is physical education a compulsory school curriculum subject in lower secondary school? | 4^th^ Worldwide Survey of Quality Physical Education | A National level experts  B - |
|  |  | In your country, is physical education a compulsory school curriculum subject in upper secondary schools? | 4^th^ Worldwide Survey of Quality Physical Education | A National level experts  B - |
| Community | - | - | - | - |
| Organisational | Average curricular physical education time per week | Within the policy framework of the compulsory school programme, how much time is allocated to the physical education curriculum each week in lower secondary schools? | 4^th^ Worldwide Survey of Quality Physical Education | A National level experts  B - |
|  |  | Within the policy framework of the compulsory school programme, how much time is allocated to the physical education curriculum each week in upper secondary schools? | 4^th^ Worldwide Survey of Quality Physical Education | A National level experts  B - |
| Interpersonal | - | - | - | - |
| Individuals | Total time spent with physical activity per week^a^ | Over the past 7 days, on how many days were you physically active for a total of at least 60 minutes per day?  Please add up all the time you spent in physical activity each day.  [0-7 days] | HBSC^c^ | A Population (adolescents)  B (13,14)  C (14) |

^a^ Only partial fit of indicator and survey question

^b^ The target group of COSI are primary school children aged 6-9 years. In some countries, children aged 11 years might attend primary school as well. Their health behaviours are assessed by the HBSC survey (see module on Physical Activity at Secondary School).

^c^ HBSC collects data on the health behaviour of children aged 11, 13 and 15 years. In some countries, children aged 11 years might still attend primary school (see module on Physical Activity at Primary School)
