# Supplementary material for: Towards a harmonized European surveillance for dietary and physical activity indicators in young and adult populations
Source: Eur J Public Health. 2022 Nov 29;32(Suppl 4):iv21–31. doi: 10.1093/eurpub/ckac061 (PMC9706124; doi:10.1093/eurpub/ckac061)
Supplement: ckac061_Supplementary_Data [file ckac061_supplementary_data.zip › ckac061_Supplementary_Data/Hebestreit_Monitoring_SupplMat4.docx]

**Supplementary Table 4: Summary of consultative process with EU surveillance systems**

| **Surveillance system** | **Willingness to implement the individual level SIMPLE questionnaire item on …** | | | | | | **Barriers (B) and Facilitators (F) /** **Additional information on implementation** |
| --- | --- | --- | --- | --- | --- | --- | --- |
|  | **Total time spent with PA** | **Walking and Cycling** | **F&V consumption** | **SSB consumption** | **Snack foods consumption** | **BMI** |  |
| **COSI** | Most likely not | No | Most likely | Most likely | In use | Yes | **B:** No WHO recommendation for leisure time PA, but only for total PA. In most European countries, leisure time PA is almost zero. IPAQ or GPAQ needed for measuring total PA but they are too extensive.  **F:** An optional sub-sample analysis with more extensive assessment in some countries would be possible.  Harmonization of COSI family questionnaire items with HBSC questionnaire is underway.  **Additional information**: Prefers to  develop and validate a single question capturing all PA domains. |
| **HBSC** | Most likely | Most likely | Most likely | In use | Most likely | Comparable instrument | **B:** Core (mandatory) questionnaire for 2021/22 was agreed upon in November 2020. Optional questionnaire adapted by the network as a whole for comparisons across countries has been also finalised.  Space limitations in questionnaires have to be considered.  **F:** Possibility to pilot test on national level and introduce the questions in an additional optional package. **Additional information:** Pilot studies will be possible in Wales and Ireland since questionnaires are not finalised for the next wave. These may offer the opportunity to discuss the modules for usage in the 2025/2026 HBSC wave. |
| **NORMO** | No | Yes  *Considers using one of the physical activity modules as an indicator for sustainability for its next wave. | Comparable instrument  *Data could be aggregated with FFQ. | Comparable instrument  *Data could be aggregated with FFQ. | Comparable instrument  *Data could be aggregated with FFQ. | Yes | **B:** Less political support as the current focus is on reducing costs and the topic sustainability, e.g. red meat consumption. Low participation rates in past waves have to be considered. Interview length should not exceed 10-15 minutes. **F:** Possibility to superimpose modules in a sub-sample (easy to reach and willing to wear the pedometers) or add questions in additional interview-administered questionnaires. If many surveillance systems agree to use the same modules and shorter instruments, it may decrease costs and increase comparability between countries. Approval by the WHO is an important aspect since it evaluates WHO recommendations and answer national health questions, e.g. calculation of trends. **Additional information:** Implementation needs to be further discussed with policy-makers. |
| **EHIS** | In use | In use | Comparable instrument | Comparable instrument  *Included in voluntary questionnaire | Comparable instrument  *Included in voluntary questionnaire | Comparable instrument | **B:** Current legal acts allow a max. 5% increase in the number of variables for EHIS wave 4 compared to wave 3. New instruments need to be broken down to the variable level. Preferable are instruments which use a minimal number of variables. It will not be possible in EHIS to ask similar questions on the same topic.  **F:** Open to discuss proposals that are first assessed by the Task Force and later proposed to an implementing regulatory body. **Additional information**: Changes in the survey require a formalised process. A Task Force will meet to discuss ideas, data analysis and evaluation. It has to be evaluated to which extent indicators from SIMPLE modules reflect or differ from EHIS questionnaire. |
| **STEPS** | No  *Includes leisure time AND transport | Comparable instrument | In use | Comparable instrument  *Using the instrument from EHIS | Comparable instrument  *With a main focus on salt intake | In use | **B:** No WHO recommendation for leisure time PA but only for total PA. In most European countries, leisure time PA is almost zero. IPAQ or GPAQ needed for measuring total PA but they are too extensive.  **F:** Possibility for an extensive assessment in optional sub-sample in some countries. |

| BMI | Body Mass Index |  |
| --- | --- | --- |
| FFQ | Food-Frequency-Questionnaire |  |
| F&V | Fruit and Vegetable |  |
| GPAQ | Global Physical Activity Questionnaire |  |
| IPAQ | International Physical Activity Questionnaire |  |
| PA | Physical Activity |  |
| SSB | Sugar-Sweetened Beverages |  |
