# Supplementary material for: Towards a harmonized European surveillance for dietary and physical activity indicators in young and adult populations
Source: Eur J Public Health. 2022 Nov 29;32(Suppl 4):iv21–31. doi: 10.1093/eurpub/ckac061 (PMC9706124; doi:10.1093/eurpub/ckac061)
Supplement: ckac061_Supplementary_Data [file ckac061_supplementary_data.zip › ckac061_Supplementary_Data/Hebestreit_Monitoring_SupplReferences.docx]

**Reference list for Tables 1&2 and Supplementary Tables 1-3**

1. Cook A, Roberts K, O'Leary F, Allman-Farinelli MA. Comparison of single questions and brief questionnaire with longer validated food frequency questionnaire to assess adequate fruit and vegetable intake. Nutrition. 2015;31(7-8):941-7.

2. Olfert MD, Barr ML, Charlier CM, Famodu OA, Zhou W, Mathews AE, et al. Self-reported vs. measured height, weight, and BMI in young adults. Int J Environ Res Public Health. 2018;15(10):2216.

3. De Rubeis V, Bayat S, Griffith LE, Smith BT, Anderson LN. Validity of self-reported recall of anthropometric measures in early life: A systematic review and meta-analysis. Obes Rev. 2019;20(10):1426-40.

4. Riordan F, Ryan K, Perry IJ, Schulze MB, Andersen LF, Geelen A, et al. A systematic review of methods to assess intake of sugar-sweetened beverages among healthy European adults and children: a DEDIPAC (DEterminants of DIet and Physical Activity) study. Public Health Nutr. 2017;20(4):578-97.

5. Vereecken CA, Maes L. A Belgian study on the reliability and relative validity of the Health Behaviour in School-Aged Children food-frequency questionnaire. Public Health Nutr. 2003;6(6):581-8.

6. WHO. The WHO STEPS Surveillance Manual 2020 [Available from: http://www.who.int/ncds/surveillance/steps/Section%203%20Show%20Cards.pdf.

7. Matthiessen J, Andersen LF, Barbieri HE, Borodulin K, Knudsen VK, Kørup K, et al. The Nordic Monitoring System 2011–2014. 2016.

8. WHO. WHO Europe Childhood Obesity Surveillance Initiative (WHO COSI): overweight and obesity among 6-9-year-old children. 2018.

9. Baumeister SE, Ricci C, Kohler S, Fischer B, Topfer C, Finger JD, et al. Physical activity surveillance in the European Union: reliability and validity of the European Health Interview Survey-Physical Activity Questionnaire (EHIS-PAQ). Int J Behav Nutr Phys Act. 2016;13:61.

10. Varnaccia G, Zeiher J, Lange C, Jordan S. Factors influencing childhood obesity - the establishment of a population-wide monitoring system in Germany Journal of Health Monitoring. 2017;2(2):85-97.

11. European Commission. Eurostat: European health interview survey (EHIS wave 2). Methodological manual. Luxembourg: European Commission2013 [Available from: <https://ec.europa.eu/eurostat/documents/3859598/5926729/KS-RA-13-018-EN.PDF/26c7ea80-01d8-420e-bdc6-e9d5f6578e7c>.

12. Leatherdale ST, Laxer RE. Reliability and validity of the weight status and dietary intake measures in the COMPASS questionnaire: are the self-reported measures of body mass index (BMI) and Canada’s food guide servings robust? International Journal of Behavioral Nutrition and Physical Activity. 2013;10(42):1-11.

13. Prochaska JJ, Sallis JF, Long B. A physical activity screening measure for use with adolescents in primary care. Arch Pediatr Adolesc Med. 2001;155(5):554-9.

14. Ridgers ND, Timperio A, Crawford D, Salmon J. Validity of a brief self-report instrument for assessing compliance with physical activity guidelines amongst adolescents. J Sci Med Sport. 2012;15(2):136-41.
